# Monomeric CRP and negative acute phase proteins, but not pentameric CRP, as biomarkers of major depression and MDMD

**DOI:** 10.1017/neu.2025.10044

**Published:** 2025-11-18

**Authors:** Abbas F. Almulla, Mengqi Niu, Drozdstoy Stoyanov, Yingqian Zhang, Michael Maes

**Affiliations:** 1 Sichuan Provincial Center for Mental Health, Sichuan Provincial People’s Hospital, School of Medicine, University of Electronic Science and Technology of China, Chengdu, China; 2 Key Laboratory of Psychosomatic Medicine, Chinese Academy of Medical Sciences, Chengdu, China; 3 Medical Laboratory Technology Department, College of Medical Technology, The Islamic University, Najaf, Iraq; 4 Department of Psychiatry, Medical University of Plovdiv, Plovdiv, Bulgaria; 5 Research Institute, Medical University of Plovdiv, Plovdiv, Bulgaria; 6 Research and Innovation Program for the Development of MU - PLOVDIV (SRIPD-MUP), Creation of a network of research higher schools, National Plan for Recovery and Sustainability, European Union – NextGenerationEU, Medical University of Plovdiv, Plovdiv, Bulgaria, Europe; 7 Department of Psychiatry, Faculty of Medicine, Chulalongkorn University, Bangkok, Thailand; 8 Kyung Hee University, Seoul, South Korea

**Keywords:** Monomeric CRP, major depression, negative acute phase proteins, inflammation, biomarkers

## Abstract

**Background:**

Contrary to the negative acute-phase protein (APP) response, there is no consistent correlation between serum pentameric C-reactive protein (pCRP) and major depression (MDD). Monomeric CRP (mCRP), a dissociation product of pCRP under immune-inflammatory conditions, exhibits pro-inflammatory effects; however, it has not been investigated in MDD or its subtypes, major dysmood disorder (MDMD) and simple dysmood disorder (SDMD).

**Objective:**

To examine serum mCRP, albumin, transferrin, M1 macrophage and Thelper-17 immune profiles, and adverse childhood experiences (ACEs) in MDD, MDMD and SDMD.

**Methods:**

Seventy-nine MDMD patients, 30 SDMD patients, and 40 controls were included. Serum mCRP was measured by ELISA; albumin, transferrin, and pCRP by biochemical assays; and cytokines using Luminex technology.

**Results:**

MDMD patients showed significantly higher mCRP compared with SDMD and controls, while both patient groups exhibited reduced albumin and transferrin. Combining mCRP with albumin and transferrin showed an adequate accuracy for MDD (area under the ROC Curve = 0.793). Adding IL-17A and ACEs improved accuracy (ROC = 0.855). Serum mCRP levels are additionally associated with pCRP, M1 macrophage profile, body mass index, and ACEs. Up to 36.6% of the variance in overall severity of depression was explained by mCRP, T-helper-17 profile, ACEs (all positively), albumin and transferrin (both inversely).

**Conclusion:**

Future research in MDD should employ mCRP rather than pCRP as a biomarker of depression/MDMD. Combining mCRP with biomarkers of the negative acute-phase response identified 63.7% of MDD patients with a smouldering acute-phase response, with a specificity of 82.1%. We recommend to assess mCRP rather than pCRP in MDD studies.

## Graphical abstract

**Figure:**
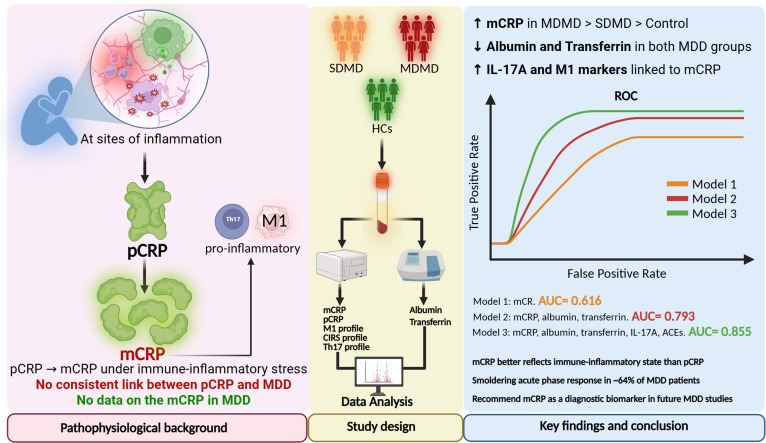

## Significant outcomes

- First study to measure serum mCRP in MDD and its subtypes
- mCRP was elevated in MDMD, while pCRP showed no change; albumin and transferrin were reduced
- A panel of mCRP, albumin, and transferrin identified MDD with AUC 0.793; adding IL-17A and ACEs raised AUC to 0.855.
- Depression severity was partly explained by mCRP and Th-17 (positive) and by albumin and transferrin (inverse).

## Limitations

- Cross-sectional design prevents causal inference
- Single-country sample limits generalizability
- Other NIMETOX domains were not assessed

## Introduction

Major depressive disorder (MDD) is increasingly identified with disruptions in neuroimmune, metabolic, and oxidative (NIMETOX) pathways (Maes et al., 2025a). MDD in its various stages is linked to the upregulated immune-inflammatory response system (IRS), particularly the M1 macrophage profile and T helper (Th)-1 and Th-17 pathways, as evidenced by increased levels of interleukin (IL)-1β, IL-6, IL-17A, and tumour necrosis factor (TNF)-α (Maes et al., 2025a). The compensatory immunoregulatory system (CIRS), which includes anti-inflammatory cytokines such as IL-10 and IL-4, as well as sIL-1RA, is frequently reduced (Maes and Carvalho, 2018; Maes et al., 2025a). The disparity between IRS and CIRS contributes to chronic inflammation and neurotoxicity, which are key characteristics of the acute phase of MDD (Maes et al., 2025a).

A precision nomothetic psychiatry approach has refined this model by classifying MDD into two primary subtypes, namely major dysmood disorder (MDMD) and simple dysmood disorder (SDMD) (Maes et al., 2022). Patients with MDMD exhibit greater severity of depression, anxiety, chronic fatigue, and suicidal behaviours, along with increased recurrence of illness (ROI) indices, in comparison to those with SDMD (Maes et al., 2022; Almulla et al., 2024a). MDMD is biologically defined by significant IRS and NIMETOX activation, which includes increased M1 macrophage and Th-1/Th-17 signalling (Maes et al., 2025a). In contrast, the initial episode of SDMD is more closely linked to CIRS inhibition (Maes et al., 2022; Maes et al., 2025d). Immune activation in first-episode MDMD is significantly associated with adverse childhood experiences (ACEs), highlighting the impact of early-life stress on immune-inflammatory dysregulation (Almulla et al., 2024b).

The acute phase response is part of IRS activation. In this process, hepatocytes elevate the levels of positive acute phase proteins (APPs), including C-reactive protein (CRP), while the levels of negative APPs, particularly albumin and transferrin, decrease (Maes, 1993; Almulla et al., 2022; Maes et al., 2025c). Reduced levels of albumin and transferrin are consistently observed in MDD and exhibit a strong correlation with immune activation, ACEs, and the severity of physio-affective symptoms (Maes, 1993; Maes et al., 2025c). Consequently, these markers offer a more direct measure of the acute phase response in MDD as compared with measurements of serum pentameric CRP (Maes et al., 2025c).

Nevertheless, pentameric CRP (pCRP) is frequently suggested as a biomarker for “inflammatory depression” when serum levels surpass 3 or 5 mg/L (Raison et al., 2013; Wessa et al., 2024; Jha et al., 2025). Certain authors have proposed utilising pCRP thresholds to inform treatment decisions regarding anti-inflammatory agents (Raison et al., 2013; Wessa et al., 2024). Nevertheless, the elevation of pCRP is more closely linked to obesity, metabolic syndrome (MetS), and ACEs than to MDD itself (Douglas et al., 2004; Moraes et al., 2017; Khan et al., 2020; Almulla et al., 2025; Maes et al., 2025c). Moraes et al. (2017) established that a large part (50%) of the variance in pCRP is determined by age, BMI and ACEs, namely sexual abuse (Moraes et al., 2017). Recently, our research indicated that in drug-naïve obese patients with MetS, depressive symptoms are not associated with pCRP but are instead related to other metabolic and immune indices (Almulla et al., 2025). In addition, Maes et al., revealed no association between MDD and pCRP, whilst lowered albumin and transferrin significantly explained MDD (Maes et al., 2025c). These findings confirm the role of negative APPs as biomarkers of MDD and pCRP as a biomarker of metabolic conditions (Almulla et al., 2025; Maes et al., 2025c).

CRP exists in various conformations, pCRP and monomeric CRP (mCRP). The latter is produced via the conformational dissociation of pCRP under certain conditions at sites of inflammation and tissue injury, where it demonstrates significant pro-inflammatory effects (Thiele et al., 2014; Olson et al., 2023). mCRP activates endothelial cells, leukocytes, and platelets, induces oxidative stress, and promotes the release of inflammatory mediators, including IL-1, IL-6, IL-8, and TNF-α (Molins et al., 2011, Ruiz-Fernández et al., 2021, Olson et al., 2023). Elevated mCRP has been associated with cardiovascular disease, Alzheimer’s disease, systemic lupus erythematosus, and hepatitis, showing stronger correlations with disease severity compared to pCRP (Jakuszko et al., 2017; Caprio et al., 2018; Hornick and Potempa, 2023; Gao et al., 2024). Due to its proinflammatory effects, mCRP has been suggested as a potential biomarker for MDD (Hornick and Potempa, 2023), however, no previous research has assessed mCRP.

This study is the first to examine serum mCRP levels in combination with pCRP, negative APPs (albumin and transferrin), M1 macrophage, Th-17 and CIRS profiles in MDMD and SDMD versus healthy controls. We will also investigate whether the integration of mCRP with negative APPs and inflammatory profiles yields enhanced diagnostic and explanatory value for distinct subtypes of MDD and severity of illness. Our hypothesis is that mCRP is significantly increased in MDD and its combination with albumin and transferrin will significantly enhance its ability to predict MDD rather than pCRP.

## Methods

### Subjects

This study included 165 participants consisting of 79 patients with MDMD, 30 with SDMD and 40 healthy controls. This case–control cross-sectional study was performed at the Psychiatric Center of Sichuan Provincial People’s Hospital, Chengdu, China. Participants were between 18 and 65 years of age, and both sexes were represented. MDD patients were diagnosed according to DSM-5, and eligibility required a Hamilton Depression Rating Scale (HAMD-21) score greater than 18 (Hamilton, 1960). Furthermore, patients were classified into MDMD (the most severe subtype) and SDMS (milder depression) based on established criteria from prior research (Maes et al., 2022). Controls were recruited from hospital staff, their relatives, and acquaintances of patients, and they were matched to cases by age, sex, education, and body mass index (BMI). Written informed consent was obtained from all participants or their legal guardians. Ethical approval was granted by the Research Ethics Committee of the Sichuan Provincial People’s Hospital [Ethics (Research) 2024-203].

In the present study, the exclusion criteria includes: (a) other psychiatric disorders rather than MDD such as schizophrenia, schizoaffective disorder, bipolar disorder, psycho-organic disorders, autism spectrum disorders, substance use disorders apart from nicotine dependence; (b) systemic medical diseases including autoimmune disorders, systemic lupus erythematosus, inflammatory bowel disease, psoriasis, type 1 diabetes mellitus, rheumatoid arthritis, chronic obstructive pulmonary disease, or cancer; (c) severe allergic reaction during the past month; (d) pregnancy or breastfeeding; (e) developmental or personality disorders, or neurological illnesses such as stroke, epilepsy, brain tumours, Parkinson’s disease, Alzheimer’s disease, or multiple sclerosis; (f) infection within the last three months; (g) current use of immunosuppressants, glucocorticoids, or other immunomodulators; (h) therapeutic doses of antioxidants or omega-3 supplements within the last three months; (i) recent surgery within the last three months; or (j) frequent use of analgesics. In addition, we excluded controls if they had a current or past diagnosis of MDD, dysthymia, DSM-IV anxiety disorders, or a family history of affective disorders, suicide, or substance use disorders (other than nicotine dependence).

### Clinical assessments

A trained physician conducted semi-structured interviews that recorded demographic data, illness course, medical and psychiatric history, and family history. Psychiatric diagnoses were confirmed using the Mini International Neuropsychiatric Interview (M.I.N.I.) (Sheehan et al., 1998), which assessed depressive episodes, dysthymia, hypomanic episodes, suicidal ideation, panic disorder, agoraphobia, social anxiety disorder, generalised anxiety disorder, obsessive–compulsive disorder, post-traumatic stress disorder, alcohol and non-alcoholic substance dependence/abuse, psychotic disorders, eating disorders, and antisocial personality disorder.

Symptom severity was assessed on the same day with the 21-item Hamilton Depression Rating Scale (HAMD) (Hamilton, 1960), the Hamilton Anxiety Rating Scale (HAMA) (Hamilton, 1959), and the State version of the State–Trait Anxiety Inventory (STAI) (Spielberger et al., 1983). A principal component (PC) was extracted from the HAMD, HAMA, and STAI values which explained 83.61% of the variance. This PC analysis showed a Kaiser-Meyer-Olkin metric of 0.727 (*p* < 0.0001), and all three scores were highly loaded on the first PC (> 0.85) with a Cronbach’s Alpha = 0.902. This PC score was employed as an overall severity of depression (OSOD) index. ACEs were assessed using the Childhood Trauma Questionnaire–Short Form (CTQ-SF) (Bernstein et al., 2003), validated in Chinese by Zhao Xingfu (Zhao et al., 2005). Emotional and physical abuse, sexual abuse, emotional neglect, and physical neglect scores were calculated following prior work (Vasupanrajit et al., 2024). Since sexual abuse appears to be a specific significant predictor of pCRP (Moraes et al., 2017) we used sexual abuse and the sum of the four other ACEs in the analyses (labelled as “sum four ACEs”).

### Anthropometrics and metabolic syndrome

Measurements included weight, height, waist circumference (WC), and BMI. The calculation of BMI involves dividing weight in kilograms by the square of height in metres. WC was taken midway between the iliac crest and the lowest rib. A composite adiposity index was computed as zBMI + zWC. Metabolic syndrome (MetS) was defined according to the 2009 Joint Scientific Statement of the American Heart Association and National Heart, Lung, and Blood Institute (Alberti et al., 2009), requiring at least three of the following: WC ≥ 90 cm in men or ≥ 80 cm in women, triglycerides ≥ 150 mg/dL, HDL-cholesterol < 40 mg/dL in men or < 50 mg/dL in women, systolic blood pressure ≥ 130 mm Hg or diastolic ≥ 85 mm Hg or antihypertensive treatment, and fasting glucose ≥ 100 mg/dL or a diagnosis of diabetes. MetS ranking was based on the number of fulfilled criteria.

### Blood collection and assays

Between 06:30 and 08:00 a.m., fasting venous blood (30 mL) was drawn into serum tubes with disposable syringes. Samples underwent centrifugation at 3500 rpm, after which serum was aliquoted into Eppendorf tubes and preserved at −80°C until analysis. mCRP concentrations were measured with a BioVendor ELISA kit (Brno, Czech Republic), sensitivity 0.63 ng/mL, intra-assay CV < 10%, and inter-assay CV < 15%. pCRP was quantified using a particle-enhanced immunoturbidimetric assay (DIAYS DIAGNOSTIC SYSTEM, Shanghai) on an ADVIA 2400 analyser (Siemens Healthcare Diagnostics Inc.). The assay had sensitivity 0.3 mg/L, intra-assay CV 2.80%, and inter-assay CV 2.17%. Serum transferrin was determined by immunoturbidimetry (DIAYS DIAGNOSTIC SYSTEM) on the ADVIA 2400, sensitivity 0.03 g/L, intra-assay CV 1.96%, and inter-assay CV 0.67%. Albumin was measured with a Bromocresol Green kit (Beijing Strong Biotechnologies, Inc.) on the ADVIA 2400, intra-assay CV 1.20% and inter-assay CV 2.10%. A negative acute-phase index was calculated as z albumin + z transferrin.

Cytokines were analysed using Luminex xMAP technology on the Luminex 200 system (Luminex Corporation, Austin, TX, USA) with the Human XL Cytokine Fixed Panel (bio-techne, R&D Systems; Cat. No. LKTM014B). Fluorescence intensity (FI) and concentrations were obtained for 46 cytokines and chemokines with FI values corrected for blanks. The protocol included: (1) dilute samples two-fold with Calibrator Diluent RD6-65; (2) add 50 μL diluted sample and 50 μL microparticle cocktail per well, shake 850 r.p.m. for 2 h at room temperature; (3) wash three times, add 50 μL Biotin-Antibody Cocktail, incubate 1 h at 850 rpm; (4) wash again, add 50 μL Streptavidin-PE, incubate 30 min at 850 rpm; (5) final wash and resuspension in 100 μL buffer for 2 min per well. The Luminex 200 quantified all analytes. Intra-assay CVs were < 5% and inter-assay CVs < 11.2%. Indices were created for immune activity: M1 macrophage activity was the z-score sum of z IL-1 + z IL-6 + z TNF-α + z sIL-1RA (Maes and Carvalho, 2018); CIRS activity was the z-score sum of z IL-4 + z IL-10 + z EGF (Maes and Carvalho, 2018); and Th-17 activity was defined as z IL-6 + z IL-17A (Maes and Carvalho, 2018).

### Data analysis

This research employed IBM SPSS for Windows, version 30, to perform all statistical analysis. Contingency tables tested categorical associations, while analysis of variance (ANOVA) compared continuous outcomes. Multiple comparisons were adjusted with the false discovery rate (FDR). Pearson correlations examined associations among continuous variables, and point-biserial correlations between continuous and binary variables. Binary logistic regression models contrasted MDD with controls, and MDMD with SDMD. Diagnosis (MDD or MDMD) served as the dependent variable, with controls or SDMD as reference. Covariates included age, sex, BMI, and smoking status. Results included Nagelkerke pseudo-R^2^, Wald statistics with p-values, odds ratios with 95% CIs, and regression coefficients (B) with standard errors (SE). The Wald statistic was defined as (B/SE)^2^. To address subgroup imbalance (SDMD and controls are underrepresented compared with MDD and MDMD, respectively), oversampling (random replicate) was applied for SDMD versus MDMD and for controls versus MDD. Cross-validated classification accuracy was tested with linear discriminant analysis and 10-fold cross-validation. Model performance was summarised with the area under the ROC (receiver operating characteristic) curve (AUC), Gini index, maximum Kolmogorov–Smirnov (Max K–S) values, and overall fit. Multiple linear regression analyses predicted acute-phase proteins or rating scale scores from demographic variables, metabolic indicators, and biomarkers including ACEs. Both manual and automated stepwise procedures were employed. Automated modelling applied entry and removal thresholds of *p* = 0.05 and *p* = 0.07, respectively. Results included standardised beta coefficients, degrees of freedom, p-values, R^2^, and F-statistics. Heteroskedasticity was tested with the White and modified Breusch–Pagan tests. Multicollinearity was checked by tolerance and variance inflation factors. All analyses were two-tailed with *α* = 0.05, and data transformations (log10, square-root, rank-order, or Winsorizing) were applied where required.

## Results

### Socio-demographic, clinical and biomarkers data

Sociodemographic and clinical characteristics for MDMD, SDMD, and controls are presented in Table 1. There were no significant differences across groups in age, sex ratio, or smoking. Metabolic variables also did not differ significantly, including MetS prevalence, MetS ranking, waist circumference, and BMI. Total HAMD, HAMA, and STAI scores were higher in MDMD than SDMD, and both patient groups scored higher than controls. Transferrin and albumin were reduced in MDMD and SDMD compared with controls, with no differences between the two patient groups. M1 macrophages were higher in MDMD than SDMD; neither patient group differed from controls. T helper-17 was elevated in MDMD compared with SDMD and controls, with no difference between SDMD and controls. The CIRS profile was increased in MDMD relative to SDMD and controls, with no difference between SDMD and controls.

**Table 1.** Socio-demographic, clinical and biomarker data of patients with major dysmood disorder (MDMD), simple dysmood disorder (SDMD) and healthy controls (HC)

Variables	Healthy Controls (*n* = 40) A	SDMD (*n* = 30) B	MDMD (*n* = 79) C	*F* value	df	*p*-value
Age (years)	37.1 (13.7)	34.9 (13.1)	35.4 (12.1)	0.32	2/146	0.728
Sex (M/F)	13/27	11/19	22/57	0.86	1	0.650
MetS (Y/N)	10/29	3/27	12/66	3.25	1	0.197
Total HAMD	0.8 (1.9) B,C	24.4 (5.0) A,C	29.9 (4.5) A,B	677.82	2/146	< 0.001
Total HAMA	1.1 (2.9) B,C	21.9 (8.3) A,C	28.1 (8.5) A,B	177.72	2/146	< 0.001
STAI, state	36.8 (8.3) B,C	54.5 (9.3) A,C	59.7 (10.6) A,B	73.58	2/146	< 0.001
Ranking MetS	1.6 (1.5)	1.3 (1.1)	1.4 (1.3)	0.45	2/144	0.636
Body mass index (kg/m2)	23.5 (4.1) C	22.0 (3.0)	22.1 (3.3) A	2.45	2/146	0.09
Waist circumference (cm)	79.4 (11.7)	79.0 (10.8)	77.2 (11.1)	0.60	2/146	0.552
Transferrin (mg/dL)	258.8 (37.4) B,C	234.7 (39.9) A	224.1 (39.9) A	10.21	2/145	< 0.001
Albumin (g/L)	43.6 (2.0) B,C	41.1 (3.4) A	41.1 (2.9) A	11.42	2/144	< 0.001
M1 macrophage (*z* score)	−0.095 (0.855)	−0.345 (1.032) C	0.203 (1.040) B	3.55	2/140	0.031
T helper-17 (*z* score)	−0.238 (0.954) C	−0.258 (0.999) C	0.272 (0.995) A,B	4.96	2/140	0.008
Interleukin-17 (*z* score)	−0.280 (0.731)	−0.032 (1.248)	0.165 (0.994)	2.51	2/140	0.085
CIRS profile (*z* score)	−0.314 (1.014) C	−0.262 (1.176) C	0.255 (0.883) A,B	5.49	2/140	0.005

Data are shown as mean (SD or SE). A,B,C, protected post-hoc comparisons (*p* < 0.05). HAMD, Hamilton Rating Scale for Depression; HAMA, Hamilton Anxiety Rating Scale; M, Male; F, Female; MetS, Metabolic syndrome; MetS, metabolic syndrome; CIRS, compensatory immune-regulatory system.

Table 2 shows no significant differences in pCRP across groups. In contrast, mCRP was higher in MDMD than controls, while SDMD and controls did not differ. In two other models, mCRP remained higher in MDMD than SDMD or controls after covarying for different explanatory variables, including pCRP, which was a significant predictor of mCRP (*F* = 12.57, df = 2/142, *p* < 0.001).

**Table 2.** Measurements of pentameric and monomeric C-reactive protein (CRP) in patients with major dysmood disorder (MDMD), simple dysmood disorder (SDMD) and healthy volunteers (HC)

Variables	HC (*n* = 40) A	SDMD (*n* = 30) B	MDMD (*n* = 79) C	*F* value	df	*p*-value
Pentameric CRP*^	0.992 (1.120)	2.488 (1.072)	2.212 (0.657)	0.288	2/142	0.592
Momomeric CRP*^	1.091 (0.069) C	1.284 (0.066)	1.410 (0.040)A	9.92	2/142	0.002
Residualized monomeric Log CRP*^	0.044 (0.017) C	0.094 (0.018)	0.129 (0.011) A	8.14	2/142	< 0.001
Residualized monomeric Log CRP**	0.050 (0.017) C	0.091 (0.017)	0.128 (0.011) A	7.50	2/142	< 0.001

All data are shown are marginal estimated mean (SE). A,B,C: protected post-hoc comparisons (*p* < 0.05). *Processed in Log10 transformation; ^, adjusted for body mass index and drug status (age and sex are not significant); ** After adjusting for body mass index, medication status, and pentameric CRP (the latter was highly significant as covariate, namely: *F* = 12.57, df = 2/142, *p* < 0.001).

### Correlations between pCRP, mCRP, metabolic indices, and ACEs

Table 3 presents Pearson’s product–moment correlations between pCRP and mCRP values and clinical measures (MetS, MetS rank, BMI, waist circumference) and psychological stressors (sexual abuse and the sum four ACEs). pCRP showed significant positive correlations with the metabolic variables and sexual abuse, but not the sum four ACEs. mCRP was significantly and positively correlated with all variables except sexual abuse. In contrast, residualized mCRP was positively correlated with the sum four ACEs.

**Table 3.** Intercorrelation matrix among pentameric and monomeric C-reactive protein (CRP), metabolic variables and adverse childhood experiences (ACEs)

Variables	Pentameric CRP	monomeric CRP	Residualized monomeric CRP^
Metabolic syndrome (MetS)	0.260** (< 0.001)	0.164* (0.037)	0.041 (0.601)
Rank MetS	0.285** (< 0.001)	0.216** (0.006)	0.059 (0.455)
Body mass index	0.380** (< 0.001)	0.249** (0.001)	0 (1)
Waist circumference	0.345** (< 0.001)	0.208** (0.008)	0.002 (0.979)
Sexual abuse	0.161* (0.039)	0.097 (0.219)	0.140 (0.074)
Sum four ACEs	0.143 (0.067)	0.194* (0.013)	0.253** (0.001)

^After adjusting for body mass index, medication status, *p* < 0.05, *p* < 0.01.

### Accuracy of pCRP, mCRP and negative APPs

Table 4 presents binary logistic regression analyses with the MDD diagnosis (healthy control as reference group) as the dependent variable and positive acute phase proteins (mCRP, pCRP), negative acute phase proteins (transferrin, albumin), and inflammatory biomarkers (IL-17, M1 macrophage) as explanatory variables. In Model #1, MDD was not significantly associated with pCRP. In Model #2, mCRP was positively associated with MDD (χ^2^ = 13.512, df = 1, *p* < 0.001; Nagelkerke = 0.072), with an overall accuracy of 60.7% (sensitivity = 62.4%, specificity = 59.0%). Model #3 shows that the residualized mCRP (adjusted for BMI and drug state) was positively associated with MDD (χ^2^ = 31.538, df = 4, *p* < 0.001; Nagelkerke = 0.168), with an accuracy of 62.8% (sensitivity = 62.5%, specificity = 63.2%). As shown in Table 5, the cross-validated accuracy was 58% with an area under the ROC curve of 0.616.

**Table 4.** Results of binary logistic regression analyses with major depression (MDD) or major dysmood disorder (MDMD) as dependent variables and controls or simple dysmood disorder (SDMD) as reference groups

Dichotomies		Explanatory Variables	*B*	SE	Wald (df = 1)	*p*	OR	95% CI
**MDD** *versus* **healthy controls**	**Model #1**	Pentameric CRP	−0.237	0.139	2.91	0.088	0.789	0.601; 1.036
	**Model #2**	Monomeric CRP	0.357	0.131	5.04	0.025	1.428	1.046; 1.950
	**Model #3**	Monomeric CRP*	0.519	0.150	11.979	< 0.001	1.681	1.253; 2.256
	**Model #4**	Monomeric CRP*	0.351	0.160	4.81	0.028	1.421	1.038; 1.945
		Transferrin	−0.532	0.167	10.16	0.001	0.588	0.424; 0.815
		Albumin	−0.857	0.159	20.56	< 0.001	0.424	0.293; 0.615
	**Model #5**	Monomeric CRP*	0.330	0.167	3.93	0.047	1.391	1.004; 1.928
		Transferrin	−0.543	0.171	10.10	0.001	0.581	0.416; 0.812
		Albumin	−0.868	0.195	19.73	< 0.001	0.420	0.286; 0.616
		Interleukin-17	0.481	0.195	6.10	0.014	1.617	1.104; 2.368
**MDMD** *versus* **SDMD**	**Model #6**	Monomeric CRP*	0.528	0.221	5.68	0.017	1.696	1.098; 2.617
	**Model #7**	Monomeric CRP*	0.795	0.272	8.52	0.004	2.215	1.298; 3.777
		M1 macrophage	0473	0.194	5.97	0.015	1.605	1.098; 2.347
		Transferrin	−0.550	0.221	6.18	0.013	0.577	0.374; :0.890

* Monomeric CRP: residualized values after adjusting for body mass index and drug status.

**Table 5.** Diagnostic and predictive performance metrics of different models in differentiating major depressed patients from healthy controls

Predictors	AUC ROC	Gini index	Max K-S	Overall Model Quality	Accuracy	Sens/Spec.
mCRP	0.616 (0.036)	0.231	0.271	0.54	58.0%	24.0%; 82.1%
mCRP, albumin, transferrin	0.793 (0.029)	0.587	0.469	0.74	72.6%	63.7%; 82.1%
mCRP, albumin, transferrin, IL-17A	0.797 (0.029)	0.594	0.507	0.74	72.6%	63.3%; 81.6%
mCRP, albumin, transferrin, IL-17A, sum four ACEs	0.855 (0.025)	0.709	0.614	0.81	77.8%	71.7%; 84.2%

mCRP, monomeric C-reactive protein (after adjusting for BMI and drug state); IL, interleukin; Sum four ACEs, adverse childhood experiences (physical and emotional abuse and neglect).

In an additional regression (Model #4), MDD was associated with the residualized mCRP values (positive), transferrin and albumin (both negative) (χ^2^ = 78.177, df = 6, *p* < 0.001; Nagelkerke = 0.379). Table 5 shows that the model accuracy was 76.1% (sensitivity = 75.8%, specificity = 76.3%), and the cross-validated accuracy was 72.6% (AUC = 0.793, Gini index = 0.587, Max K-S = 0.469). Analysis of the coordinates of the ROC curve showed that using these three APPs, 63.7% of the MDD patients were correctly classified with a specificity of 82.1%. Model #5 indicates that residualized mCRP and IL-17A (both positive), together with transferrin and albumin (both negative), are associated with MDD (χ^2^ = 78.177, df = 6, *p* < 0.001; Nagelkerke = 0.062), with a cross-validated accuracy of 72.6% (AUC = 0.797, Gini index = 0.594, Max K-S = 0.507, see Table 5). Adding ACEs to the previous model resulted in a cross-validated accuracy of 77.8% (AUC = 0.855, Gini index = 0.709, Max K-S = 0.614).

Table 3 also reports two models distinguishing MDMD (SDMD as reference group) using mCRP, M1 macrophage, and transferrin as predictors. Model #6 shows a positive association between mCRP and MDMD (χ^2^ = 6.526, df = 1, *p* = 0.011; Nagelkerke = 0.062), with an accuracy of 55.4% (sensitivity = 36.7%, specificity = 80.0%). Model #7 shows a significant association of mCRP and M1 (both positive) and transferrin (negative) with MDMD (χ^2^ = 24.968, df = 3, *p* = 0.011; Nagelkerke = 0.226), yielding an accuracy of 63.7% (sensitivity = 82.7%, specificity = 40.0%).

### Prediction of the severity of depression

Table 6 summarises the multivariate regression analyses in which pCRP, mCRP, and OSOD were used as dependent variables, with demographic data, negative APPs, ACEs, and inflammatory biomarkers entered as explanatory variables.

**Table 6.** Results of multiple regression analysis with serum C-reactive protein (CRP) and severity of depression score as dependent variables and biomarkers and clinical data as explanatory variables

Dependent Variables	Explanatory Variables	Coefficient statistics			Model statistics			
		*β*	*t*	*p*	R^2^	*F*	df	*p*
**#1. Pentameric CRP**	**Model**				0.263	10.85	5/152	<0.001
	Body mass index	0.349	4.83	<0.001				
	Sexual abuse	0.138	1.85	0.066				
	M1 macrophage profile	0.344	3.69	<0.001				
	CIRS profile	−0.241	−2.62	0.01				
	Sum four ACEs	0.156	2.09	0.039				
**#2. Monomeric CRP**	**Model**				0.16	9.78	3/154	<0.001
	Body mass index	0.268	3.55	<0.001				
	Sum four ACEs	0.244	3.28	0.001				
	M1 macrophage profile	0.176	2.34	0.02				
	Pentameric CRP	0.219	2.89	0.004				
**#3. Overall severity of depression**	**Model**				0.366	16.48	5/143	<0.001
	Sum four ACEs	0.376	5.32	<.001				
	Albumin	−0.177	−2.4	0.018				
	Monomeric CRP	0.184	2.69	0.008				
	Transferrin	−0.184	−2.58	0.011				
	T helper-17	0.136	2.02	0.046				

CIRS, compensatory immune-regulatory system; Sum four ACEs, adverse childhood experiences (physical and emotional abuse and neglect).

Regression model #1 shows that 26.3% of the variance in pCRP was explained by BMI, sexual abuse, M1 macrophage, and four ACEs (all positive associations), together with CIRS (negative association). Regression model #2 demonstrates that 16% of the variance in mCRP was explained by BMI, four ACEs, M1 macrophage, and pCRP (all positive associations). Regression model #3 indicates that 36.6% of the variance in the OSOD score was explained by sum four ACEs, Th-17 profile, and mCRP (positive associations), along with albumin and transferrin (negative associations).

## Discussion

### Elevated mCRP in MDD

This study is the first to quantify serum mCRP in MDD. The first major finding of this study is that MDMD patients display significantly higher mCRP levels compared with SDMD and controls, while SDMD does not differ from controls. These differences remained significant after adjustments for BMI, medication status, age, and sex. In contrast, pCRP exhibited no differences between groups, corroborating previous studies that did not identify elevated pCRP (either raw or adjusted values) in MDD (Maes et al., 1992; Maes et al., 2025c). The current study confirmed that, in addition to mCRP elevations, there were reductions in the negative APPs, specifically albumin and transferrin, among patients with MDD subtypes, thereby supporting the role of the negative APP response (Maes et al., 2025c). This is consistent with earlier research indicating lower levels of albumin and transferrin in MDD (Ambrus and Westling, 2019; Gregg et al., 2020; Al-Marwani et al., 2023) and meta-analytic data showing diminished albumin levels in MDD and bipolar disorder (Almulla et al., 2022). The immune-inflammatory changes were most significant in MDMD, which exhibited greater mCRP levels, M1 macrophage activation, elevated Th-17 levels, and increased CIRS activity relative to SDMD. Previously, it was shown that MDMD, in contrast to SDMD, is the MDD phenotype that associates with immune activation (Maes et al., 2022; Almulla et al., 2024a).

Using mCRP as a solitary biomarker distinguished MDD patients from controls, though with modest accuracy (58%). Incorporating albumin and transferrin raised accuracy above 72%, while the addition of serum IL-17A produced additional gains. By contrast, pCRP had no discriminatory value (Almulla et al., 2025; Maes et al., 2025c). These findings show that mCRP has greater relevance than pCRP, but negative APPs remain the strongest biomarkers of the immune-inflammatory response in MDD or SDMD.

The current study highlights that mCRP is most effective as part of a biomarker panel for MDD when combined with other immune-inflammatory markers such as serum albumin, transferrin and IL-17A. Likewise, a large part of the variance in OSOD was explained by the regression on mCRP (not pCRP), the negative APPs and IL-17A. Past work demonstrated a significant accuracy for negative APPs in melancholia, with sensitivity of 72% and specificity of 92% (Maes et al., 1991; Maes, 1995). These data align with recent findings confirming serum IL-17A as a biomarker of MDD (Maes and Carvalho, 2018; Moulton et al., 2024) although other studies reported limited or sex-specific effects (Saraykar et al., 2018; Xie et al., 2023; Tsuboi et al., 2024).

IL-17A was identified in the present study as an independent predictor of MDMD severity, suggesting it accounts for unique variance beyond the overall Th-17 profile. The Th-17 composite indicates a coordinated pro-inflammatory pathway that includes IL-6 and IL-17A (Almulla et al., 2024a). However, the distinct effect of IL-17A implies its central and non-redundant role in maintaining the chronic low-grade inflammation typical of MDMD. This supports the perspective that IL-17A serves not only as a pathway marker but also as a potential therapeutic target in the Th-17 axis.

In contrast, Wessa et al., claimed that pCRP ≥ 3.0 mg/L defines “inflammatory depression” in about 30% of patients (Wessa et al., 2024). Our data refute this stance, showing that pCRP is not even increased in MDD and, as explained below, is a metabolic biomarker. Even more important, employing mCRP coupled with negative APPs and IL-17A identifies around 63.7% of MDD patients with a reasonable accuracy. Thus, the conclusion that 30% of MDD patients show “inflammatory MDD” is inadequate because around 63% of those patients show an immune profile reminiscent of a smouldering immune response. In this respect, a recent study showed that 78.8% of MDD patients exhibit at least one NIMETOX abnormality (Maes et al., 2025b). Furthermore, if one considers that the other phenotype of MDD, namely SDMD, often exhibits suppression of CIRS activities, one may conclude that most MDD patients, if not all, show phenotype-specific immune alterations. Thus, stratifying patients using this inadequate pCRP criterion and using this as an indicant to treat MDD with anti-inflammatory medications (Wessa et al., 2024) is fundamentally inaccurate.

### Determinants of CRP isoforms

The second major finding of this study indicates that mCRP is predicted by pCRP, BMI, the M1 macrophage profile and ACEs. In contrast, pCRP was predicted by BMI, ACEs, and M1 macrophages (positive) and CIRS (negative) profiles. As reviewed in the introduction, pCRP dissociates in serum into mCRP during increased inflammatory and oxidative load. This may also explain the difference between both CRP isoforms in their association with different immune profiles. Thus, while pCRP was associated with the M1 macrophage profile, which is known to induce the positive AP response in the liver, it was also inversely associated with the CIRS profile, which is known to inhibit the AP response (Maes et al., 2025c). Other predictors of both pCRP and mCRP encompassed metabolic indices, including BMI, waist circumference and MetS ranking. Nevertheless, the relationship between pCRP and metabolic variables were more pronounced than those with mCRP. The findings support the notion that pCRP in the lower concentration ranges (1 – 10 mg/L, (Almulla et al., 2025) is indicative of metabolic dysregulation rather than immune-inflammation processes associated with depression (Almulla et al., 2025; Maes et al., 2025c). Importantly, this study found that the association between mCRP and depression (diagnosis or severity) was independent from metabolic variables and pCRP. These findings indicate that mCRP, in contrast to pCRP, is a disease-specific biomarker of MDD and MDMD.

Both serum pCRP and mCRP were significantly predicted by ACEs. The latter also impacted the negative APP response (Maes et al., 2025c), reflecting their broader impact on NIMETOX pathways (Maes et al., 2025a). As a consequence, ACEs play a critical role in the AP response established in MDD or MDMD. Prior studies showed that ACEs elevate CRP and MetS risk later in life (Danese et al., 2007; Danese et al., 2009; Lin et al., 2016; Iob et al., 2020; Balaji and Sankaranarayanan, 2023; Zagaria et al., 2024, O’Shields et al., 2025). Furthermore, the accuracy for MDD improved when ACEs were included along with mCRP and both negative APPs.

### mCRP as an inflammatory biomarker

mCRP enhances immune-inflammatory processes via various mechanisms. For example, mCRP promotes platelet aggregation, vascular activation, enhanced expression of adhesion molecules on endothelial cells, neutrophil migration, NK-cell activity, and activation of the complement system (Molins et al., 2011; Zeinolabediny et al., 2021; Lazarut-Nistor and Slevin, 2025). These actions promote thrombosis and vascular inflammation, phenomena also observed in MDD (Tonhajzerova et al., 2020). Concurrently, mCRP enhances the expression of IL-6, IL-8, TNF-α, VCAM-1, COX-2, and MMP-13 via NF-κB signalling and promotes the generation of reactive oxygen species (Ruiz-Fernández et al., 2021, Olson et al., 2023). Moreover, mCRP interacts with key immune pathways thereby activating microglia and inflammasomes via iNOS, COX-2, and NLRP3, thereby amplifying neuroinflammation in brain tissues (Bartra et al., 2025). The observed effects align closely with the immune-inflammatory, M1 macrophage and Th-17 patterns identified in MDMD (Maes et al., 2022; Almulla et al., 2024a), offering substantial evidence that mCRP plays a role in the immune signature associated with MDD and MDMD (Hornick and Potempa, 2023).

In adaptive immunity, mCRP interacts with cholesterol in CD4+ T cell membranes, modifying TCR conformation to a primed state that increases IFN-γ release (Zhou et al., 2023). It indirectly activates T cells via monocytes by upregulating CD80, which subsequently engages CD80/CD28 co-stimulation. This pathway is inhibited by Belatacept, thereby affirming its specificity (Thomé et al., 2025). Furthermore, neurodegenerative and autoimmune models highlight the pathogenic potential of mCRP. In Alzheimer’s disease, mCRP inhibits ApoE expression and disrupts ApoE–LRP1 binding, consequently heightening neuronal vulnerability (Na et al., 2023). In stroke models, mCRP is identified in endothelial cells and neurons within peri-infarct regions, associated with toxic inflammatory and angiogenic responses (Slevin et al., 2010; Lazarut-Nistor and Slevin, 2025). In lupus nephritis, immune complexes formed between mCRP and anti-mCRP antibodies intensify inflammation, interacting with anti-dsDNA and anti-C1q antibodies (Sjöwall et al., 2003, Trouw et al., 2004, Jönsen et al., 2007, Jakuszko et al., 2017). The findings suggest that mCRP may have detrimental effects as a neurodegenerative and inflammatory driver.

Interestingly, diminished albumin resulting from the adverse acute phase response may cause elevated mCRP levels. Albumin serves as the primary plasma transporter for lysophosphatidylcholine (LPC), which has cytotoxic properties and activates the immune response. Consequently, reduced albumin levels may increase free LPC bioavailability, facilitating the dissociation of pCRP into mCRP (Durço et al., 2023).

## Limitations

This research presents several limitations. The cross-sectional design limits the ability to draw causal conclusions regarding the relationships among elevated mCRP, immune activation, negative APPs, and the severity of depression. Longitudinal investigations are required to determine whether mCRP changes precede, coincide with, or follow the clinical course of MDD. Second, the study population consisted exclusively of Chinese patients with MDD, which may limit the generalizability of the findings to other ethnic or cultural groups. Replication in diverse populations is therefore essential. Third, although mCRP was examined in combination with immune-inflammatory markers, future research should also evaluate its interaction with additional NIMETOX components, particularly oxidative and nitrosative stress pathways, and atherogenicity profiles (Maes et al., 2025a). Depression is frequently linked to a ‘leaky gut’ (Maes et al., 2025a), which plays a role in hepatocyte CRP production, gut dysbiosis, and reductions in short-chain fatty acid levels, particularly butyrate (Pant et al., 2023). Butyrate exerts significant suppressive effects on immune-inflammatory processes and systemic mitochondrial function (Anderson and Maes, 2020). Future research should clarify the role of gut-associated changes in the pathophysiological measures of this study.

The limited clinical viability of measuring mCRP represents an additional limitation. The assay remains limited in availability and standardisation within conventional laboratory environments, despite its considerable scientific importance. The application is confined to research laboratories because of the associated costs and the requirement for specialised reagents and techniques. Future research should focus on developing mCRP assays that are cost-effective, validated, and standardised to facilitate broader clinical application. Finally, this study discussed mechanisms that may be induced by mCRP, such as NF-κB and NLRP3 activation; however, we did not directly assess these intracellular pathways in patients with MDD. Therefore, while our cytokine and acute-phase protein profiles correspond with this activation, these mechanisms remain inferential and require validation through targeted molecular analyses in MDD-derived samples.

## Conclusion

This work provides the first evidence that serum mCRP is significantly elevated in MDD, most notably in MDMD, independent of BMI, medication status, and pCRP. The association of mCRP with M1 activation, Th-17 signalling, and reduced negative APPs defines a distinct immune-inflammatory phenotype in MDD largely overlapping with MDMD. These findings demonstrate that mCRP is more relevant than pCRP for characterising MDD/MDMD and support its integration into precision psychiatry approaches.

## Data Availability

The corresponding author (MM) will provide access to the dataset supporting this study upon reasonable request, subsequent to a comprehensive data review.
